# Peptide YY in Type 2 Diabetes: A Complementary Gut Hormone with Therapeutic Potential Beyond GLP-1

**DOI:** 10.3390/nu17213468

**Published:** 2025-11-03

**Authors:** Nhi Thi Nguyen, Jae-Hyung Park

**Affiliations:** Department of Physiology, Keimyung University School of Medicine, Daegu 42601, Republic of Korea; nguyenthinhiydh@gmail.com

**Keywords:** gut hormone, peptide YY, GLP-1, incretin, type 2 diabetes, satiety, bariatric surgery, β-cell preservation, combination therapy

## Abstract

Type 2 diabetes (T2D) is a complex metabolic disease characterized by insulin resistance, progressive β-cell dysfunction, and persistent hyperglycemia. While GLP-1 receptor agonists have revolutionized the management of T2D by improving glycemic control and reducing body weight, their insulinotropic effects increase the workload on pancreatic β-cells, which may hasten β-cell decline in certain individuals. Peptide YY (PYY), a gut-derived hormone secreted alongside glucagon-like peptide-1 (GLP-1) from L-cells, presents a unique and complementary therapeutic approach. In contrast to GLP-1, PYY does not directly induce insulin release but confers metabolic advantages by suppressing appetite through Y2 receptor pathways, enhancing insulin sensitivity via peripheral Y1/Y4 receptors, and slowing gastric emptying to minimize postprandial glucose surges. Notably, recent research suggests PYY supports the preservation and restoration of pancreatic islets by improving their structure and function without increasing the secretory demand. PYY levels are substantially increased after bariatric surgery, where it plays a pivotal role in weight-loss-independent improvements in glycemic regulation and islet hormone dynamics. These attributes position PYY as a strong candidate for use in combination with GLP-1 analogs, especially in individuals with advanced β-cell impairment or those who respond inadequately to GLP-1 monotherapy. This review discusses PYY’s physiological functions, mechanistic actions, and therapeutic opportunities in T2D, highlighting its potential as a valuable adjunct or alternative in gut-hormone-oriented treatment strategies.

## 1. Introduction

Type 2 diabetes (T2D) continues to pose a significant global health threat, which is largely attributable to increasing obesity prevalence and widespread sedentary behaviors, which constitute major risk factors for onset [1]. T2D is generally defined by a triad of insulin resistance, progressive pancreatic β-cell failure, and sustained hyperglycemia [1]. Despite the availability of multiple classes of pharmacologic agents, consistently achieving and maintaining both optimal glycemic control and weight reduction remains problematic, emphasizing an urgent need for innovative treatments that address the multiple pathogenic mechanisms of T2D [1]. Therapies that target incretin pathways, particularly those aimed at the glucagon-like peptide-1 (GLP-1) axis, have significantly advanced the management of diabetes by supporting better glycemic outcomes, facilitating weight reduction, and delivering cardiovascular risk reduction [2]. As an example, GLP-1 receptor agonists such as semaglutide and liraglutide are highly recommended in international guidelines based on robust evidence of their effectiveness in lowering HbA1c by around 1.0–1.5% and reducing body weight by approximately 5–15% [3].

Alongside the clinical success of GLP-1 agonists, other gut-derived peptides such as peptide YY (PYY) offer additional therapeutic opportunities, particularly for patients who do not achieve optimal outcomes with GLP-1 therapies [1]. PYY, which is co-released with GLP-1 by L-cells in the distal intestine in response to nutrient ingestion, demonstrates circulating concentrations that increase in direct correlation with caloric intake [4,5]. Notably, evidence indicates that PYY interacts with multiple neuropeptide Y receptors (Y1, Y2, Y4, Y5). This peptide primarily modulates appetite and energy balance through Y2 receptor-mediated suppression within the hypothalamus [5,6]. Additionally, preclinical studies have shown that PYY can improve insulin sensitivity via peripheral Y1 receptors and delay gastric emptying, thereby attenuating postprandial glycemic excursions [1]. Although PYY does not exert a direct insulinotropic effect—a critical difference compared to GLP-1—its multifaceted actions on satiety, alimentary behavior, and glucose regulation underscore its potential for addressing both overeating and hyperglycemia in T2D patients [1].

Early studies revealed that circulating levels of the anorexigenic peptide PYY_3–36_ are diminished in overweight and obese individuals, suggesting that impaired PYY signaling contributes to hyperphagia and metabolic dysfunction. These observations provided the first rationale for exploring PYY as a therapeutic target for obesity and diabetes, a line of investigation that has since expanded to include its roles in insulin sensitivity and β-cell preservation [7,8].

This review evaluates the physiological significance of PYY in glucose homeostasis, synthesizing current data from preclinical and clinical T2D research, and drawing direct comparisons with GLP-1 regarding receptor selectivity, metabolic efficacy, and adverse event profiles. In addition, we discuss the translational prospects of PYY-centered therapeutic strategies, either as monotherapy or as adjuncts to established incretin-based regimens, highlighting their promise in preserving β-cell reserve and optimizing long-term glycemic management in T2D.

## 2. Physiology of PYY and GLP-1

### 2.1. Peptide YY (PYY)

Peptide YY (PYY) is a 36-amino acid peptide hormone predominantly released by enteroendocrine L-cells of the ileum and colon in response to the ingestion of nutrients, with enhanced secretion following intake of fat and protein [1]. PYY circulates in two bioactive isoforms: PYY_1–36_ and PYY_3–36_, with PYY_3–36_ being the principal form in the bloodstream due to processing by dipeptidyl peptidase-4 (DPP-4) [2]. PYY_3–36_ mediates its appetite-suppressing effects via selective activation of hypothalamic Y2 receptors located in the arcuate nucleus (ARC), resulting in inhibition of orexigenic neuropeptide Y (NPY) and agouti-related peptide (AgRP) neurons, while indirectly activating pro-opiomelanocortin (POMC) neurons [3]. Consequently, PYY is effective in suppressing caloric intake and plays a key role in promoting postprandial satiation [4]. Additionally, PYY delays gastric emptying and reduces intestinal motility, thereby enhancing nutrient absorption and prolonging satiety sensations [5]. Furthermore, data from preclinical models and select human studies indicate that PYY contributes to improved insulin sensitivity, decreased food intake, and lowered body weight. For instance, Hoek et al. found that administration of PYY significantly reduced food consumption in mice maintained on a high-fat diet, supporting its proposed role in the regulation of energy homeostasis [9]. In a separate investigation, Batterham et al. demonstrated that PYY is essential for mediating protein-induced satiation, underscoring its importance in body weight regulation [10]. In addition, Zhou et al. provided evidence on the physiological involvement of PYY, showing that fluctuations in PYY levels can profoundly affect appetite regulation and energy balance [11].

However, it is essential to recognize that the mechanism of PYY action differs from that of other incretin hormones, such as GLP-1. This important distinction must be considered because, while GLP-1 stimulates insulin secretion, PYY predominantly exerts its effects by enhancing satiety without directly promoting insulin release [12,13,14].

### 2.2. Glucagon-like Peptide-1 (GLP-1)

GLP-1, a 30-amino acid incretin hormone generated from the proglucagon gene, is secreted by the same L-cell population in response to carbohydrate and fat ingestion [15]. It is rapidly degraded by DPP-4, resulting in a brief half-life of roughly 2 min; nevertheless, longer-acting GLP-1 receptor agonists have been engineered for therapeutic use [3]. The physiological actions of GLP-1 are mediated by the GLP-1 receptor (GLP-1R), which is abundantly expressed in pancreatic β-cells, the central nervous system, the gastrointestinal tract, and cardiovascular tissues [16]. Importantly, GLP-1 has been shown to provide multiple benefits, including stimulation of glucose-dependent insulin secretion, inhibition of glucagon secretion, slowing of gastric emptying, and reduction in appetite through central pathways [17]. In addition to its insulin-promoting properties, GLP-1 supports β-cell survival and may help maintain β-cell mass across various preclinical studies [18]. In contrast to PYY, GLP-1 has been evaluated extensively in large clinical studies and serves as the foundation for several approved treatments for T2D and obesity, such as liraglutide and semaglutide. These pharmaceuticals reliably lower HbA1c, promote weight loss, and deliver cardiovascular benefits [3].

## 3. Role of PYY in Glucose Homeostasis

While PYY has long been recognized for its role in energy balance and appetite regulation, recent research demonstrates that it also participates in the regulation of glucose homeostasis through several mechanisms [19]. Although PYY does not function as an insulinotropic hormone like GLP-1, it has been linked to improved glycemic control, primarily by modulating insulin sensitivity, suppressing appetite, and influencing gastrointestinal motility [20]. More specifically, PYY complements GLP-1 by increasing insulin sensitivity and decreasing food intake without directly stimulating insulin secretion [21]. PYY can decrease stress on pancreatic β-cells by minimizing postprandial blood glucose variability and increasing insulin sensitivity independently of insulin secretion, whereas GLP-1 primarily contributes to postprandial blood glucose management through increased insulin release [22]. The well-established clinical benefits of GLP-1 raise the question of whether PYY-based interventions might offer similar therapeutic effects. To further explore this possibility, the following sections will present a detailed comparison of PYY and GLP-1 in relation to their roles in glucose and energy metabolism (Table 1).

### 3.1. Preclinical Evidence

A substantial and growing body of preclinical evidence has examined the metabolic actions of PYY, particularly the PYY_3–36_ isoform, in rodent models of obesity and insulin resistance. Almost all studies report that PYY_3–36_ exhibits both anti-obesity and insulin-sensitizing properties, making it a promising candidate for T2D management [27]. In diet-induced obese (DIO) mice, chronic peripheral administration of PYY_3–36_ leads to significant reductions in food intake, body weight, and fat mass [28]. The underlying central mechanisms are thought to involve the activation of Y2 receptors located in the ARC of the hypothalamus [29]. Through direct engagement of this receptor, PYY may induce hyperpolarization in NPY and AgRP neurons and decrease neuronal firing rates [30,31]. Additionally, PYY may improve insulin sensitivity by acting via Y1 and Y4 receptors in peripheral tissues such as the liver, adipose tissue, and islets, highlighting its broader metabolic impact beyond the regulation of appetite [28].

Beyond that, the beneficial impact of PYY_3–36_ on glucose metabolism has also been documented. In DIO mice, daily subcutaneous administration of PYY_3–36_ for 2–4 weeks led to significant improvements in fasting glucose concentrations, glucose tolerance, and insulin sensitivity, as determined by glucose and insulin tolerance assessments [27]. In leptin-deficient (ob/ob) and leptin receptor-deficient (db/db) mice, PYY treatment promoted insulin signaling cascades in skeletal muscle and liver, as indicated by elevated phosphorylation levels of insulin receptor substrate (IRS)-1 and AKT [32]. Furthermore, administration of PYY_3–36_ suppressed hepatic gluconeogenesis, in part by downregulating critical gluconeogenic enzymes, including phosphoenolpyruvate carboxykinase (PEPCK) and glucose-6-phosphatase (G6Pase) [33]. There is also evidence that PYY elicits peripheral insulin-sensitizing actions. Specifically, in adipocytes and myocytes, PYY_3–36_ treatment increased insulin-stimulated glucose uptake, which may be attributed to enhanced insulin receptor signaling and activation of AMP-activated protein kinase (AMPK) [34]. Collectively, these observations indicate that the glucose-lowering effects of PYY operate through additional mechanisms beyond central appetite control. Notably, PYY knockout mice exhibit features of metabolic impairment, characterized by elevated food consumption, increased body weight, and compromised glucose tolerance, reinforcing the physiological significance of PYY in maintaining metabolic homeostasis [35].

In summary, preclinical studies support the hypothesis that PYY—especially PYY_3–36_—facilitates a diverse range of beneficial effects on both energy balance and glucose metabolism via central and peripheral pathways (Table 2). Nevertheless, it is important to exercise caution when extrapolating these findings to humans, given that interspecies variation in gut hormone signaling and receptor distribution may affect potential therapeutic efficacy.

### 3.2. Human Studies

While preclinical research consistently shows metabolic benefits of PYY, evidence from human studies is more limited and sometimes contradictory. Multiple clinical studies have investigated PYY’s involvement in glucose metabolism, appetite regulation, and insulin sensitivity among individuals with and without obesity or T2D [37]. Diminished postprandial PYY responses have been observed in people with obesity and T2D. For example, plasma PYY levels after consuming a mixed meal are significantly reduced in subjects with T2D compared to non-diabetic controls, indicating a diminished L-cell response or disrupted gut hormone signaling in diabetes [38]. This reduction may contribute to impaired satiety signaling and increased caloric intake, ultimately worsening insulin resistance and hyperglycemia. Importantly, Roux-en-Y gastric bypass (RYGB) and sleeve gastrectomy procedures result in a marked and sustained elevation of postprandial PYY levels—frequently surpassing pre-surgical values—which is associated with increased satiety and improved metabolic outcomes [39,40]. Collectively, these findings indicate that boosting endogenous PYY secretion might underlie some of the metabolic benefits seen after weight loss surgery [37].

Short-term administration of PYY_3–36_ in healthy volunteers has consistently demonstrated a dose-dependent decrease in appetite and caloric consumption, typically between 20% and 30% in controlled feeding experiments, which supports its appetite-suppressing actions mediated through the hypothalamic Y2 receptor pathway [41]. Conversely, the impact of PYY on glucose regulation in humans is less definitive. For example, in a double-blind crossover trial, infusion of PYY_3–36_ in lean and obese participants produced only modest reductions in postprandial glucose and insulin, and these changes were less pronounced than those achieved with GLP-1 [42]. Other investigations, however, have found no significant shifts in glucose or insulin despite suppression of appetite, highlighting considerable variability in PYY’s metabolic effects depending on individual factors and experimental design [43].

Few studies have evaluated the effects of chronic PYY administration in humans, mainly because of pharmacokinetic challenges such as rapid degradation and tolerability issues, particularly the prevalence of nausea at elevated doses [44]. Early-phase clinical trials utilizing continuous infusion or long-acting analogs have produced inconsistent outcomes: certain studies reported short-term reductions in body weight and improvements in insulin sensitivity, whereas others failed to observe sustained metabolic benefits, possibly attributable to patient habituation, suboptimal drug exposure, or adverse effects that restrict therapeutic dosing [45]. At present, no PYY-based therapy has advanced to late-phase clinical evaluation, and there is no definitive evidence supporting its efficacy in glycemic control when used alone in humans [46]. Recent investigations with long-acting PYY analogs (e.g., Y14, CIN-110) have yielded improved pharmacokinetic characteristics. In Phase 1 trials, Y14 administered subcutaneously at specified intervals led to durable reductions in body weight and decreased caloric intake [26,47]. Although mild nausea was present, repeated dosing did not result in tachyphylaxis. CIN-110, now undergoing Phase 1 evaluation, displays greater receptor selectivity and molecular stability specifically designed to address PYY’s limited half-life and tolerability challenges. Collectively, these analogs signify meaningful advances in the development of chronic PYY-based therapeutic strategies [48,49].

Across available human studies, PYY administration consistently induces marked reductions in appetite and caloric intake, accompanied by delayed gastric emptying and lower postprandial glucose excursions. However, improvements in overall glycemic control and insulin sensitivity have been modest and variable across trials. Acute infusion studies in overweight and obese individuals demonstrated a clear suppression of energy intake and a slight reduction in postprandial glucose and insulin responses, but no sustained changes in fasting glucose or HOMA-IR indices were observed [7,50]. In early phase trials of long-acting PYY analogs, moderate weight reduction and enhanced satiety were achieved, whereas glycemic improvement remained secondary and limited [26]. When PYY was co-administered with GLP-1, additive or synergistic effects on satiety and caloric restriction were observed without additional gastrointestinal adverse events [50,51]. Collectively, current human data suggest that while PYY monotherapy provides robust appetite suppression and gastric slowing, its direct metabolic benefit for glycemia and insulin sensitivity is limited; nevertheless, combination approaches with GLP-1 hold translational promise for enhanced metabolic control with improved tolerability.

A concise overview of representative preclinical and early human studies investigating PYY and its combination with GLP-1 is provided in Table 3. This table summarizes the model characteristics, dosing regimens, exposure durations, and principal metabolic outcomes across translational stages.

## 4. Mechanistic Insights of PYY

### 4.1. Central Appetite Regulation and Energy Balance

One of the best-studied effects of PYY_3–36_ is its anorexigenic property, which is mainly exerted via the hypothalamic ARC. Following nutrient ingestion, PYY_3–36_ is released into the circulation and traverses the blood–brain barrier, selectively interacting with Y2 receptors (Y2R) [51]. This receptor engagement initiates a β-arrestin signaling cascade in NPY/AgRP neurons, predominately operating via the Gi pathway, and leads to inhibition of these neurons, which are strong promoters of appetite [23,54]. At the same time, by suppressing this orexigenic pathway, there is an indirect enhancement of POMC neuron activity, which advances satiety through melanocortin signaling, particularly via MC4R activation [51]. The result is appetite suppression, earlier cessation of eating, and reduced overall caloric intake. In animal models, intracerebroventricular administration of PYY_3–36_ produces marked reductions in food consumption, and this effect is abolished by genetic ablation or pharmacological inhibition of Y2 receptors, underscoring the essential role of Y2R signaling [51]. Human functional neuroimaging has shown that exogenous PYY reduces activation in brain regions related to food reward and incentive motivation, such as the orbitofrontal cortex and striatum [55]. The long-term consequences of this appetite reduction can be clinically meaningful. Persistent decreases in energy intake may promote body weight loss, which subsequently enhances insulin sensitivity, diminishes hepatic steatosis, and alleviates systemic inflammation—key pathophysiological mechanisms driving insulin resistance in T2D [56].

In addition, the action of PYY through the gut–brain axis provides a distinct therapeutic benefit: in contrast to conventional appetite-suppressing agents that often exert widespread effects on the central nervous system, PYY targets specific satiety mechanisms and typically does not cause compensatory increases in food intake or metabolic disturbances when imitating physiological postprandial signals. Nonetheless, an important challenge is the short half-life of endogenous PYY and its derivatives, warranting the development of delivery modalities that can prolong or target drug action to uphold effective appetite suppression without eliciting nausea or adverse reactions [57].

L-cell secretion of gut hormones is influenced not only by nutrient-derived signals but also by autonomic inputs. β-adrenergic stimulation of L-cells increases intracellular cAMP and promotes GLP-1 and PYY release, as shown in rat ileal perfusion studies [58]. In contrast, α_2_-adrenergic signaling exerts an inhibitory effect, reducing hormone secretion under conditions of elevated sympathetic tone [59,60]. Together, these findings indicate that the sympathetic nervous system exerts bidirectional control over L-cell activity, coordinating the secretion of GLP-1 and PYY according to physiological context.

### 4.2. Delayed Gastric Emptying and Glucose Absorption

Current evidence indicates that PYY has a crucial function in regulating gastrointestinal motility, mainly by slowing gastric emptying and small intestinal transit—effects that are important for postprandial glucose homeostasis. After food intake, PYY is released from L-cells in the distal gut and signals through enteric neurons and vagal afferents, leading to the activation of a “colonic brake” mechanism that decreases upper gastrointestinal motility [29]. The PYY_3–36_-mediated delay in gastric emptying results in a slower and more controlled delivery of nutrients to the small intestine, thereby moderating the postprandial rise in blood glucose by reducing the rate of glucose absorption. Animal experiments have confirmed this pathway; however, among the cited literature, there is no direct acute human evidence demonstrating that PYY prolongs gastric emptying and reduces glycemia.

On a mechanistic level, PYY is thought to exert its effects primarily by binding to Y2 receptors expressed on enteric neurons, where it suppresses excitatory cholinergic transmission within the gastrointestinal system [29]. Central signaling pathways involving vagal afferents to the brainstem and hypothalamus are also involved in modulating gastric tone and motility [61]. In people with obesity or type 2 diabetes, secretion of PYY after meals is frequently diminished, which may account for the observed acceleration of gastric emptying and increased blood glucose variability [62]. Enhancing endogenous PYY activity or using pharmacological agents that mimic PYY signaling may, therefore, represent a promising therapeutic strategy by stabilizing postprandial glucose levels—a primary objective in diabetes care, especially for individuals at elevated cardiovascular risk [63].

Although GLP-1 receptor agonists are established medications that delay gastric emptying and lower postprandial blood glucose, the addition of PYY has been shown in experimental studies to exert complementary or synergistic effects when used alongside GLP-1 [64]. These findings imply that co-treatment with PYY and GLP-1-based agents could provide superior glycemic control, potentially allowing for dosage reductions and a lower incidence of adverse effects like nausea [36].

### 4.3. Peripheral Insulin Sensitivity

Beyond its central anorexigenic properties and modulatory influence on gastrointestinal motility, PYY—particularly the PYY_3–36_ isoform—may improve peripheral insulin sensitivity by both direct and indirect mechanisms. The exact pathways have not yet been fully elucidated, but early preclinical data implicate PYY in the regulation of insulin action within primary metabolic sites such as skeletal muscle, adipose tissue, and the liver [9]. In obese and insulin-resistant rodent models, sustained administration of PYY_3–36_ is linked to better insulin sensitivity, as evaluated by insulin tolerance and hyperinsulinemic-euglycemic clamp methods [9]. These metabolic benefits seem to arise independently of weight reduction and are supported by both central signals and peripheral actions.

In skeletal muscle, PYY_3–36_ has been demonstrated to enhance insulin-stimulated glucose uptake, likely through upregulation of insulin signaling pathways, including increased phosphorylation of IRS-1 and AKT, which are critical components of the insulin signaling cascade [8]. In adipose tissue, PYY may facilitate lipid mobilization and indirectly improve insulin sensitivity by mitigating inflammation and reducing adipocyte hypertrophy—both of which are characteristic features of insulin resistance [65]. In the liver, PYY administration has been associated with decreased hepatic steatosis and downregulation of gluconeogenic enzymes (e.g., PEPCK and G6Pase), indicating potential enhancements in hepatic insulin responsiveness [8].

Although the precise receptor-mediated pathways in peripheral tissues are not yet fully characterized, multiple mechanisms have been suggested. Y2 receptor expression has been detected in peripheral tissues, which supports the possibility of PYY exerting direct hormonal effects outside the central nervous system [52]. Another proposed mechanism is that PYY acts centrally to suppress sympathetic nervous system activity, resulting in improved insulin sensitivity and glucose uptake in peripheral tissues through changes in autonomic regulation. Additionally, PYY may provide anti-inflammatory benefits that facilitate insulin signaling at the cellular level [66].

Evidence regarding the effect of PYY on peripheral insulin sensitivity in humans remains sparse and inconsistent. Small-scale studies in overweight or obese populations have reported that acute infusion of PYY_3–36_ can lead to modest improvements in oral glucose tolerance and Homeostatic Model Assessment of Insulin Resistance (HOMA-IR) [67]. Nevertheless, these outcomes are not consistently replicable, and data on long-term effects are absent [68]. Notably, the magnitude of insulin sensitivity improvement may depend on the metabolic status of individuals, with pronounced benefits observed among those with underlying metabolic dysfunction and insulin resistance [69]. Furthermore, individual differences in PYY responsiveness—potentially due to variations in receptor sensitivity, gut microbiota composition, or genetic background—may influence its overall metabolic impact [70].

### 4.4. Insulin and Glucagon Secretion

PYY is a pleiotropic gut hormone involved in the modulation of glucose metabolism, encompassing both insulin and glucagon secretion. While animal studies provide valuable mechanistic insights, clinical research increasingly suggests a role for PYY in human T2D pathophysiology. Earlier investigations, exemplified by the work of Khan et al., demonstrated that PYY can impact islet hormone secretion and pancreatic islet structure, although these data are predominantly derived from in vitro and animal models Khan et al. [25]. Tan et al. found that administration of PYY_3–36_ in DIO mice led to improved fasting glucose and insulin sensitivity without affecting body weight, though it is crucial to account for interspecies differences when translating these findings to humans [53].

Mahat et al. examined the direct actions of PYY on islet receptors (e.g., Y1, Y4), indicating the potential for broader metabolic effects beyond appetite control [71]. Nevertheless, Boey et al. demonstrated an absence of both acute and chronic insulinotropic actions of PYY across multiple mouse models, highlighting significant variability in PYY responsiveness that may be influenced by differences in receptor distribution and species-specific factors [65]. These divergent findings imply that PYY’s influence on islet hormone secretion may be contingent on contextual factors such as disease status and duration of exposure.

Additionally, Knop et al. reported that both fasting and postprandial PYY concentrations were markedly reduced in individuals with T2D compared to non-diabetic controls. Reduced PYY levels were associated with elevated fasting glucose and insulin levels, supporting a potential link between disrupted PYY secretion and impaired islet function as well as defective incretin responses in T2D cohorts [72].

## 5. Comparison of PYY and GLP-1 in the Context of Type 2 Diabetes

PYY and GLP-1 are gastrointestinal hormones that are co-released from enteroendocrine L-cells in response to nutrient ingestion. While GLP-1 has established incretin activity and significant therapeutic benefits for T2D, the clinical utility of PYY continues to be explored. Their similar secretion patterns, together with different receptor targets and physiological effects, suggest they may have complementary roles in metabolic homeostasis.

### 5.1. Secretion and Postprandial Dynamics

Both PYY and GLP-1 are secreted by L-cells located mainly in the distal ileum and colon, with nutrient intake—especially meals that are high in fat and protein—inducing their release into the circulation [5]. Despite sharing a short half-life due to rapid degradation by DPP-4, advanced pharmacological preparations of GLP-1 receptor agonists have addressed this limitation. In comparison, stable and well-tolerated methods for administering PYY are still being developed [51].

Although arising from the same L-cells, PYY and GLP-1 operate through distinct receptor mechanisms and regulate separate physiological domains. GLP-1 engages a single, well-defined receptor, GLP-1R, predominantly present in pancreatic β-cells, the gastrointestinal tract, central nervous system, and cardiovascular tissues. It promotes glucose-dependent insulin secretion, suppresses glucagon output, slows gastric emptying, and decreases food intake—all in a glucose-dependent fashion. PYY, particularly its PYY_3–36_ form, acts selectively on the Y2 receptor. In the hypothalamus, activation of the Y2 receptor inhibits orexigenic neurons (NPY/AgRP), thereby suppressing appetite [50]. Peripheral Y1 and Y4 receptors may have supplemental functions in modulating insulin sensitivity and islet hormone output, but these effects are less consistent and may vary with metabolic conditions [73]. Notably, GLP-1 possesses direct insulinotropic properties, whereas PYY primarily affects glycemic regulation by modulating appetite and, in select contexts, through enhancement of peripheral insulin sensitivity [24].

### 5.2. Therapeutic Implications and Synergistic Potential

The distinct mechanisms of action exhibited by GLP-1 and PYY have led to growing interest in employing combination therapies that target several metabolic pathways simultaneously. GLP-1 primarily exerts effects on pancreatic function, while PYY is influential in appetite control; together, these complementary actions may yield additive or synergistic therapeutic benefits. Evidence from preclinical models reveals that combined administration of PYY_3–36_ and GLP-1 results in greater reductions in both food intake and body weight compared to the effects seen with either agent alone [74]. Data from clinical research indicate that dual infusion strategies can improve satiety and decrease energy intake without corresponding increases in adverse gastrointestinal effects, such as nausea [5]. Ongoing developments in polyagonist pharmacology include the design of novel molecules or treatment approaches that recapitulate the integrated hormonal responses observed postprandially in the gut, with simultaneous targeting of PYY, GLP-1, and additional peptides (such as GIP and oxyntomodulin) [75]. Collectively, these results highlight that, while GLP-1 remains central in incretin-based interventions, PYY has potential as an adjunct in multi-targeted therapeutic approaches for optimizing metabolic control in T2D.

Recent clinical investigations reinforce the synergistic therapeutic value of PYY/GLP-1 co-administration. Notably, a Phase 2 clinical trial examining NNC0165-1875, a PYY/GLP-1 polyagonist, demonstrated superior outcomes for both glycemic control and body weight reduction compared to monotherapies, while also showing a reduction in gastrointestinal adverse events [76,77]. Other reports suggest that a Y2-selective PYY analog (NNC-1273) exhibits improved efficacy in lowering glucose when paired with GLP-1 agonists over single-agent therapy [78,79]. These cumulative findings support the clinical promise of multi-hormone strategies for achieving lasting glycemic improvements in T2D management.

### 5.3. Clinical Positioning and Candidate Populations

PYY-based interventions may offer distinct advantages for specific patient populations with type 2 diabetes (T2D) who exhibit heterogeneity in response to incretin-based therapy. Individuals who show suboptimal glycemic or weight responses to GLP-1 receptor agonists (GLP-1RAs) represent a key candidate group [2]. In such patients, the addition of PYY could enhance satiety and slow gastric emptying without further increasing β-cell secretory demand [50].

Patients with advanced T2D or reduced β-cell reserve may also benefit from PYY’s non-insulinotropic mechanism, which helps preserve islet function by lowering postprandial glucose excursions and reducing β-cell stress [52]. Conversely, in early-stage or obesity-driven T2D characterized by hyperphagia and rapid gastric emptying, PYY may complement existing incretin therapy through appetite control and improved insulin sensitivity at peripheral sites [26].

From a translational perspective, combination strategies leveraging both GLP-1 and PYY could enable lower dosing of each hormone, thereby mitigating gastrointestinal side effects such as nausea while maintaining efficacy in appetite and glucose control [50,51]. Preliminary co-infusion trials in humans have shown additive effects on satiety and caloric intake reduction without excess adverse events [50,51]. As such, PYY-targeted or dual-agonist formulations may hold clinical promise for patients who are GLP-1 non-responders, have limited β-cell capacity, or require appetite suppression with minimal insulinotropic stress. Further large-scale, long-term trials are warranted to clarify which metabolic phenotypes derive the greatest benefit from PYY-based therapy.

## 6. Therapeutic Potential of Peptide YY: Insights from Bariatric Surgery

### 6.1. Post-Bariatric Diabetes Remission and Hormonal Modulation

Recent progress in metabolic surgery has questioned the traditional assumption that T2D is an inexorably advancing disease. Specifically, RYGB has been linked to persistent remission of hyperglycemia in a substantial subset of patients, frequently manifesting within a few days postoperatively and preceding meaningful weight loss [80]. This rapid normalization of glucose levels implies that factors apart from caloric restriction and weight reduction are instrumental in the improvement of glucose regulation after bariatric surgery. Among various gut-derived hormones involved, PYY has been recognized as a principal regulatory factor. Increases in postprandial PYY levels are observed following RYGB and sleeve gastrectomy, with sustained elevation observed over the long term [81]. Enhanced satiety and better glycemic regulation have been correlated with this postoperative increase in PYY [82].

Notably, research has demonstrated that elevated circulating PYY following RYGB can positively influence insulin and glucagon secretion in isolated islets from patients with T2D, independent of changes in body weight [83]. This suggests that PYY may serve as a crucial mediator in the improvement of glycemic control after surgery. Additional studies have provided supporting evidence that increased levels of PYY post-surgical intervention recalibrate appetite regulation and result in improved metabolic parameters [84]. Given the growing focus on the role of gut hormones in T2D management, exploring the clinical application of PYY alongside current therapeutic modalities presents a promising direction for future research.

### 6.2. PYY and the Restoration of Islet Function

PYY has been demonstrated to enhance islet architecture, attenuate β-cell stress, and reestablish insulin-glucagon homeostasis in diabetic models—effects that are mechanistically distinct from the insulinotropic actions of GLP-1 and indicate direct protective roles at the level of the endocrine pancreas. To facilitate a clearer understanding of PYY’s unique properties, the major differences between these two peptides are briefly outlined in Table 4. In rodent models of diabetes, administration of exogenous PYY_3–36_ has resulted in greater glucose-stimulated insulin secretion and suppression of excess glucagon release [25]. Evidence from Khan et al. further shows that PYY has an overall favorable impact on islet function, with key roles in regulating insulin secretion and promoting cell survival, which corresponds with improved glucose homeostasis [25]. Additionally, the work of Guida et al. demonstrated that treatment with PYY_3–36_ in streptozotocin (STZ)-induced diabetic rats led to significant reductions in blood glucose and preservation of histological indicators of β-cell health [52].

Emerging evidence from β-cell-ablated and type 1 diabetes (T1D) models provides additional insight into PYY’s regulatory roles in islet homeostasis. In STZ-induced diabetic rats, chronic PYY_3–36_ administration reduced fasting glucose and preserved residual islet structure despite severe β-cell depletion [52]. Intra-islet analyses further showed that PYY directly suppresses glucagon release via Y_1_-receptor-mediated autocrine/paracrine signaling in α-cells, aiding restoration of the insulin-to-glucagon ratio [67]. Complementary experimental work indicates that pancreatic/islet PYY is widely expressed (predominantly in α-cells) and is functionally important for insulin secretion and β-cell viability, supporting a mechanistic basis for islet peptide cross-talk [25,85]. These findings, together with broader reviews of PYY’s islet actions, suggest a multifaceted role for PYY in maintaining islet integrity and glucagon control even in β-cell–deficient states, although direct confirmation in human T1D remains limited [86].

In clinical research, Guida et al. observed that intra-islet PYY expression was elevated following RYGB surgery and this change correlated with enhanced β-cell responsiveness, emphasizing a regulatory function for PYY in islet health [52]. Mechanistic studies have further elucidated that PYY may exert its effects through multiple Y receptors—primarily Y1 and Y4 subtypes—localized on islet cells. Findings from Lafferty et al. indicate that activation of Y1 receptors influences islet performance by increasing insulin output and supporting β-cell viability in various metabolic states [87]. Collectively, these observations redefine PYY as a hormone with significant endocrine and paracrine activity in the regulation of glycemia, surpassing its previously established role in satiety [55].

**Table 4:** Effects of PYY on Islet Health and β-cell Function.

Effect	Description	Mechanism	Comparison to GLP-1	Refs.
Islet Architecture	Improves structural integrity of pancreatic islets	Enhances islet cell composition and cohesion	GLP-1 may preserve β-cell mass but primarily enhances insulin secretion	[25,52]
β-cell Stress	Reduces oxidative and metabolic stress on β-cells	Indirect insulin demand reduction via appetite suppression	GLP-1 augments insulin secretion, which may increase β-cell workload	[8,52]
Hormone Balance	Restores the insulin-to-glucagon ratio	Regulates intra-islet signaling through Y receptors	GLP-1 suppresses glucagon and promotes insulin secretion via GLP-1R	[25,67]
Functional Recovery	Improves both glucose-stimulatedinsulin and glucagon secretory responses	Enhances β-cell responsiveness through paracrine mechanisms	GLP-1 directly promotes GSIS through the cAMP/PKA signaling pathway	[67,87]
Islet Preservation	Protect and preserve β-cell function in diabetes models	Chronic PYY exposure associated with improved islet histology	GLP-1 may preserve β-cell mass but primarily enhances secretion	[52,87]

Evidence derived from STZ and diet-induced diabetic rodent models; human confirmation from RYGB and ex vivo islet studies. GSIS, glucose-stimulated insulin secretion.

### 6.3. PYY in the Context of Bariatric Mimicry and Combination Therapies

Given the consistent post-surgical elevation of PYY, considerable research has aimed to pharmacologically reproduce this hormonal response. Camacho-Ramírez et al. demonstrated that PYY is crucial for the improvements in glycemic control observed after RYGB in rats; specifically, pharmacological inhibition of PYY negated the metabolic benefits of the surgery [88]. In parallel, the simultaneous administration of GLP-1 and PYY_3–36_ has led to more pronounced reductions in food intake and superior metabolic outcomes compared to either hormone alone [89]. The development of multiagonist peptide therapies—including those combining GLP-1, GIP, and PYY actions—represents an approach to replicate the complex hormonal milieu post-RYGB. These compounds have shown enhanced weight loss, increased insulin sensitivity, and improved β-cell preservation in preclinical animal models [90].

Beyond pancreatic effects, PYY has also been implicated in metabolic adaptations involving other organs. For example, Pérez-Arana et al. found that PYY acts as a mediator for GLP-2-dependent intestinal hypertrophy following RYGB, a process that may contribute to improved nutrient absorption and overall energy homeostasis [89]. A recent clinical trial by Kowalka et al. demonstrated that the secretion of both PYY_1–36_ and PYY_3–36_ is significantly higher after RYGB when compared to sleeve gastrectomy, with this elevation correlating to enhanced postprandial glycemic control [91].

The current focus in multiagonist drug development is on incorporating PYY to more closely replicate the hormonal changes following bariatric surgery. Polyagonists that co-target PYY, GLP-1, and GIP receptors have led to significantly better β-cell preservation in primate studies, yielding an 18% greater reduction in HbA1c compared with GLP-1 therapies alone [49,90]. These agents recapitulate the endocrine adaptations seen after RYGB by simultaneously enhancing satiety (through PYY), increasing insulin secretion (through GLP-1), and optimizing nutrient handling (through GIP), identifying them as promising new “bariatric mimetic” agents [49,92].

Although PYY plays an important physiological role, the advancement of PYY-based pharmacotherapies presents notable obstacles. As a peptide with a brief half-life of about four minutes, PYY exhibits limited bioavailability unless delivered using advanced methods [93]. Studies testing continuous infusion or nasal spray delivery of PYY have so far demonstrated only limited success in early clinical phases [94]. Moreover, administration of high doses of PYY_3–36_ has produced significant dose-limiting adverse events, such as nausea, likely due to activation of Y2 receptors in the brainstem [95].

Receptor heterogeneity further complicates the development of novel therapeutics. PYY acts on at least four Y receptor subtypes (Y1, Y2, Y4, Y5), which exhibit distinct patterns of expression across both central and peripheral tissues [43]. The creation of receptor-selective agonists that specifically target islet-relevant pathways, while minimizing off-target effects, remains a major challenge. However, the integration of PYY into multi-target strategies presents considerable potential. Co-formulation with GLP-1 or SGLT2 inhibitors has the potential to reduce required doses, improve tolerability, and more effectively address the multifactorial nature of T2D compared to monotherapy. Nevertheless, PYY monotherapy has been hampered by limited glucose-lowering efficacy (HbA1c reduction < 0.5% in trials) and persistent nausea at effective doses [96].

Combination strategies may help overcome these challenges: co-formulation with GLP-1 enables the use of lower PYY doses while preserving therapeutic effects. Still, optimizing receptor selectivity (Y2 versus Y1/Y4) to minimize emetic side effects and establishing sustained efficacy in human populations are critical obstacles. Active clinical investigations of agents like CIN-110 and NNC0165-1875 will help determine the utility of PYY in polyhormonal regimens [49]. Combining PYY with GLP-1 analogs may yield additive metabolic advantages, especially for patients with impaired β-cell function or those who do not achieve adequate glucose control on GLP-1 monotherapy.

PYY has gained prominence as a possible mediator of diabetes remission after bariatric surgery, with emerging evidence implicating it in hormonal regulation, appetite modulation, and broad metabolic adaptations [97]. Development of PYY-based monotherapies is still at an early stage, but the hormone’s broad functional capacity and synergy with established agents like GLP-1 position it as a promising candidate for future combination regimens. Advancement of PYY-oriented interventions will require the development of highly selective receptor modulators, innovative drug delivery methods, and well-designed clinical trials to assess sustained metabolic benefit in human subjects [98].

## 7. Conclusions

PYY, a gut-derived hormone that has traditionally been investigated for its anorexigenic functions, is now increasingly recognized for its diverse contributions to glucose regulation and overall metabolic homeostasis. Although GLP-1 remains central to T2D therapy due to robust insulinotropic and cardiometabolic properties, PYY offers an additional therapeutic profile with hypothalamic appetite inhibition, delayed gastric emptying, enhanced insulin sensitivity, and possible islet-preserving effects.

A comparative analysis of PYY and GLP-1 highlights both their common physiological origins and their differing, receptor-specific mechanisms of action. While GLP-1 acts primarily through a single receptor pathway with pronounced effects on pancreatic function, PYY engages multiple NPY receptors and exhibits a wider range of central and peripheral activities. These mechanistic differences provide a strong foundation for pursuing their combined or synergistic application in multi-hormone therapeutic approaches.

Emerging clinical and experimental evidence indicates that PYY plays a pivotal role in mediating the metabolic improvements observed after bariatric surgery, especially Roux-en-Y gastric bypass. Persistent postoperative increases in PYY levels have been linked to greater satiety, enhanced glycemic regulation, and, notably, the restoration of insulin and glucagon secretion from impaired pancreatic islets. These results imply that PYY may be involved in not only the acute regulation of energy intake but also in facilitating long-term modification of disease processes in T2D.

Although preclinical studies and mechanistic findings are encouraging, PYY-based therapies are still at an early stage of clinical development. There are outstanding challenges related to peptide instability, limited receptor specificity, and suboptimal tolerability, which must be addressed by advanced formulation methods and more selective receptor-targeted drug designs. However, integrating PYY into multi-agonist strategies—especially in conjunction with GLP-1 or GIP—represents a compelling direction. Animal studies indicate that coadministration of PYY and GLP-1 analogs enhances both satiety and glycemic outcomes, allowing for decreased doses and mitigating adverse effects such as nausea. Given its non-insulinotropic mechanism, PYY could serve as a beneficial adjunct for patients inadequately managed with GLP-1–based treatments, including those experiencing significant β-cell impairment.

In conclusion, PYY is a physiologically relevant and mechanistically adaptable candidate for T2D therapy. With expanding insight into its involvement in islet regeneration and post-surgical metabolic adaptation, PYY has the potential to transition from an experimental satiety agent to an essential component of advanced diabetes therapies focused on restoring endocrine capacity and enabling lasting glycemic control.

## Figures and Tables

**Table 1:** Comparative Overview of PYY and GLP-1 in Glucose and Energy Homeostasis.

Feature	Peptide YY (PYY)	Glucagon-Like Peptide-1 (GLP-1)	Refs.
Primary Source	L-cells of distal small intestine and colon	L-cells of distal small intestine and colon	[4,5]
Main Active Form	PYY_3–36_ (via DPP-4 cleavage)	GLP-1_7–36_ (active form; rapidly degraded by DPP-4)	[2,7]
Main Receptor(s)	Y2 (central), Y1 and Y4 (peripheral)	GLP-1 receptor (GLP-1R)	[2,23]
Central Action	Appetite suppression via Y2 receptor in hypothalamus	Appetite suppression via GLP-1R in hypothalamus and brain stem	[6,24]
Peripheral Action	Enhances insulin sensitivity via Y1/Y4 receptors in liver, muscle, and adipose tissue	Promotes insulin secretion via GLP-1R in pancreatic β-cells	[9,17]
Insulinotropic Effect	Does not cause direct stimulation of insulin secretion	Has a robust glucose-dependent insulinotropic response	[8,16]
Effect on β-cells	Preserves islet architecture and mitigates β-cell stress	Stimulates insulin secretion and may promote β-cell mass expansion	[17,25]
Effect on Gastric Emptying	Delays gastric emptying	Delays gastric emptying	[3,12]
Clinical Application	Currently under investigation (PYY_3–36_, CIN-110, etc.)	GLP-1RAs approved for clinical use (liraglutide, semaglutide, etc.)	[2,26]

Values derived from both animal and human studies; n ranges from 6–30 (rodent) and 8–30 (human). DPP-4, dipeptidyl peptidase-4. BW, body weight.

**Table 2:** Mechanisms of PYY Action on Target Organs.

Therapeutic Target	Receptor	Mechanism	Metabolic Effects	Refs.
Hypothalamus (Arcuate Nucleus)	Y2	Inhibits NPY/AgRP neurons Indirectly activates POMC neurons	Appetite ↓ Satiety ↑	[6,19]
Stomach GI Tract	Y2	Inhibits cholinergic signaling in enteric neurons Modulates vagal feedback	Gastric emptying ↓ Glucose absorption ↓	[12,36]
Skeletal Muscle	Y1, possibly Y4	Enhances insulin signaling via IRS-1 and AKT phosphorylation	Glucose uptake ↑ Insulin sensitivity ↑	[9,27]
Liver	Y1	Suppresses gluconeogenic enzymes (PEPCK, G6Pase)	Hepatic glucose production ↓	[11,27]
Adipose Tissue	Y1, possibly Y4	Improves insulin receptor signaling and induces AMPK activation	Insulin sensitivity ↑ Lipogenesis ↓	[9,28]

Mechanisms primarily established in rodent and cell-based models; confirmed partially in human tissues. IRS, insulin receptor substrate; AMPK, AMP-activated protein kinase.

**Table 3:** Summary of Key Preclinical and Early Human Studies on PYY.

Stage	Model	Agent & Dose	Duration	Primary Endpoints	Headline Outcome	Refs.
Preclinical	DIO mice	PYY_3–36_ (s.c.)	2–4 wks	Food intake, BW, GTT/ITT	Intake/BW ↓ Glucose tolerance ↑ Insulin sensitivity ↑	[9,14]
Preclinical	ob/ob, db/db mice	PYY_3–36_	days–weeks	Insulin signaling (IRS-1/AKT), gluconeogenesis	Insulin signaling ↑ (muscle/liver) PEPCK/G6Pase ↓	[11,27]
Preclinical	STZ rat (β-cell ablated)	PYY_3–36_	weeks	FPG, islet histology	Glycemia ↓ Preserved β-cell architecture	[52]
Human (acute)	Lean/obese (crossover)	PYY_3–36_ infusion	hours	Energy intake, postprandial glucose/insulin	Intake 20–30% ↓ Glycemia ↓ (modest)	[7,50]
Human (Phase 1)	Overweight/obesity	Long-acting PYY analog (e.g., Y14)	weeks	BW, caloric intake, safety	BW ↓ Intake ↓ Mild nausea PK improved	[26]
Human (acute)	Overweight/obese	PYY + GLP-1 co-infusion	hours	Satiety/energy intake; insulin dynamics	Additive satiety Early insulin response ↑	[51,53]

Animal studies n = 6–15 per group; human acute studies n = 8–20, exposure 2–6 h; Phase 1 trials 4–12 weeks. BW, body weight; GTT, glucose tolerance test; ITT, insulin tolerance test; FPG, fasting plasma glucose; PK, pharmacokinetics.

## Data Availability

No new data were created or analyzed in this study. Data sharing is not applicable to this article.

## Abbreviations

The following abbreviations are used in this manuscript:

T1D	Type 1 diabetes
T2D	Type 2 diabetes
PYY	Peptide YY
GLP-1	Glugagon-like peptide-1
GLP-1R	GLP-1 receptor
NPY	Neuropeptide Y
AgRP	Agouti-related peptide
POMC	Pro-opiomelanocortin
DIO	Diet-induced obese
IRS	Insulin receptor substrate
PEPCK	Phosphoenolpyruvate carboxykinase
G6Pase	Glucose-6-phosphatase
AMPK	AMP-activated protein kinase
RYGB	Roux-en-Y gastric bypass
ARC	Arcuate nucleus
Y2R	Y2 receptor
DPP-4	Dipeptidyl peptidase-4
GSIS	Glucose-stimulated insulin secretion
STZ	Streptozotocin
